# On-site manual therapy for firefighters in Seoul: six-month utilization and outcome from a retrospective service analysis and web-based survey

**DOI:** 10.1186/s12998-026-00640-4

**Published:** 2026-04-17

**Authors:** Sungmin Park, Bo-Hyoung Jang

**Affiliations:** 1 Seoul Korean Medicine Association, Seoul, Korea; 2 Health Promotion Division, Deogyang-Gu Public Health Center, Goyang, Korea; 3 Department of Preventive Medicine, College of Korean Medicine, Kyung Hee University, 26 Kyungheedae-Ro, Dongdaemun-Gu, Seoul, 02447 Korea

**Keywords:** Musculoskeletal pain, Manual therapy, Firefighters, On-site clinic, Occupational health

## Abstract

**Background:**

In 2023, firefighters in Seoul responded to over two million emergency calls, exposing them to substantial physical and psychological strain. This working environment increases the risk of musculoskeletal disorders (MSDs) and occupational stress. To address these issues, the Seoul Metropolitan Fire & Disaster Headquarters (SMFDH) piloted a manual therapy program provided at fire stations. To our knowledge, this manuscript is the first report describing the feasibility of such an intervention in fire stations.

**Methods:**

From 11 June to 6 December 2024, one Korean Medicine (KM) doctor rotated through five fire stations, providing free one-on-one treatment sessions during duty hours. This retrospective report analyzed detailed clinical records regarding manual therapies. Baseline characteristics, diagnoses according to ICD-10, and visit frequency were compared between firefighters with ≤ 10 years and > 10 years of service. A web-based survey (12–18 November 2024) assessed self-reported pain reduction and satisfaction.

**Results:**

A total of 376 firefighters received 808 sessions; 66.8% had a history of herniated disc or rotator cuff syndrome. Manual therapies—primarily myofascial release, joint mobilization, and muscle energy techniques—were applied in 94.8% of visits. No serious adverse events were reported. Among 148 survey respondents (39.4%), self-reported NRS pain scores were lower after the program in most cases (82.4%). Firefighters with longer tenure reported a lower proportion of pain improvement but higher satisfaction scores than those with shorter tenure. Most respondents (97.3%) recommended the service, with on-site accessibility as the key advantage.

**Conclusions:**

Station-based manual therapy appeared feasible and well accepted, with participants reporting pain reduction and high satisfaction. Their working tenure influenced utilization and perceived benefit. In 2025, SMFDH extended the program to a nine-month schedule across fifteen stations. Controlled studies with objective functional outcomes and long-term follow-up are warranted to confirm its occupational health benefits.

**Supplementary Information:**

The online version contains supplementary material available at 10.1186/s12998-026-00640-4.

## Background

In Korea, firefighters have diverse duties, such as fire suppression, emergency medical services (EMS), rescue teams, and other civil services. These roles make them vulnerable to repetitive handling of heavy equipment, lifting and transporting patients during EMS duties, and prolonged rescue operations in awkward postures. The 24-h rotating duty system further poses barriers by creating scheduling challenges for timely healthcare access [1, 2]. In Seoul, 7434 firefighters responded to 2,029,547 emergency calls in 2023, illustrating the heavy workload. Firefighters’ physically demanding and high-risk tasks predispose them to musculoskeletal disorders (MSDs), as well as respiratory and psychological conditions [4–6].

MSDs are particularly common, yet firefighters often accept pain as normal and forgo treatment because of demanding work shifts and peer pressure [7, 8]. The International Association of Fire Fighters has acknowledged the benefits of early physical interventions for injured firefighters [9]. However, in contrast to this perspective, a 2025 review of Korean firefighter studies found that the majority (77.1%) focused on psychological and social interventions rather than addressing MSDs [10]. To improve access to MSD care, on-site delivery of Traditional, Complementary, and Integrative Medicine (TCIM) services may offer a practical solution [11].

TCIM’s accessible modalities—such as manual therapies and acupuncture—are supported by clinical evidence for the treatment of musculoskeletal pain [12–15]. Thus, the Seoul Metropolitan Fire & Disaster Headquarters (SMFDH) introduced on-site KM clinics at fire stations in 2024 [16]. Operated by the Seoul Korean Medicine Association (SKMA), this program aimed to relieve musculoskeletal pain by providing on-site manual therapies [17]. As medical expenses are reimbursed under government-funded policies [18], SMFDH prioritized delivering services directly at fire stations rather than relying on reimbursement-based support. By operating on-site clinics, the program sought to enhance accessibility by reducing schedule disruptions and peer pressure.

Building on this initiative, this study reports the six-month pilot program delivering on-site manual therapy for firefighters in Seoul. We collected both clinical and patient-reported data to capture feasibility and outcomes. While manual therapy is a core component of TCIM [19, 20], its application has rarely been studied for high-risk occupations. This study aimed to evaluate the feasibility, utilization patterns, and self-reported outcomes of a station-based manual therapy program for firefighters. In the long run, the research may inform practical strategies for integrating TCIM into occupational health services for high-risk worker populations.

## Methods

### Participants

With the approval of the Kyung Hee University Institutional Review Board (No. KHSIRB-25-05-EA), this study analyzed two sources of data: clinical records and survey responses from the on-site manual therapy program. From June 11 to December 6, 2024, SMFDH operated the on-site KM services for its firefighters. Each day, up to twelve firefighters received free one-on-one clinical sessions. Among five stations (Dongdaemun, Gangdong, Gwangjin, Songpa, and Jungbu), firefighters who expressed interest were scheduled on a rotating weekly basis. For Gwangjin (with two firehouses) and Jungbu (with four firehouses), on-site KM services were dispatched to all of their affiliated firehouses for three weeks at each site. To ensure accessibility while minimizing work disruption, appointments were scheduled at each fire station on a first-come, first-served basis.

### Intervention

Each session began with a standardized physical evaluation—range of motion testing and functional status measures—to guide a personalized treatment plan. Manual therapies, including myofascial release (MFR), muscle energy techniques (MET), high-velocity, low-amplitude (HVLA) thrusts, and joint mobilization techniques were administered according to protocols from the Korean Society of Chuna Manual Medicine for Spine & Nerves (KSCMM) [21]. These manual protocols are aligned with basic training and safety standards set by the International Federation for Manual/Musculoskeletal Medicine (FIMM) [22].

The KM doctor completed the 27th regular workshop of the Korean Society of Chuna Manual Medicine for Spine & Nerves (KSCMM), a program consisting of 120 h of manipulation training, including test-based evaluation. In addition to this program, he had already accumulated extensive clinical experience with more than 3,000 patients and over 10,000 treatment sessions across five years. Each KM clinic session, held weekly, lasted approximately 30 min and was provided on an individual basis. For manual therapies, the KM doctor preferred soft-tissue techniques over spinal manipulation to minimize risk of adverse events. All adverse events were monitored and documented after each session. No serious adverse events were reported during the six-month program.

All clinical interventions were conducted on a multipurpose electric bed (B1870G; Goodpl), which was pre-installed at each participating fire station by the SMFDH for this pilot project. To facilitate myofascial release, a percussive device (TheraGun Elite, 4th Generation; Therabody Inc) was employed. For primary KM therapies, acupuncture needles (0.25 × 30 mm and 0.25 × 40 mm; KMS) were used, along with two types of cups (45 × 65 mm and 23 × 65 mm; Dongbang Medical) for cupping treatments. Acupuncture and cupping were selectively applied to patients with limb joint pain or stress-related complaints. Normally, these modalities were used exclusively in sessions without manual therapies, primarily due to time constraints.

### Data collection

Clinical records of 376 firefighters from five stations were collected. Medical history, physical examination, and counseling were conducted at the initial stage. After de-identification, the corresponding author conducted descriptive analyses, focusing on utilization patterns and treatment delivery rather than clinical effectiveness. These records were stratified by tenure (≤ 10 vs. > 10 years), since longer service duration (> 10 years) is positively associated with musculoskeletal occupational disorders [23, 24]. We did not stratify by gender, given the predominance of male firefighters [3]. According to the Regulation on Position Assignment and Personnel Exchange of Fire Officials [25], firefighters rotate through administrative and field roles every 3–5 years; therefore, tenure was considered a more stable indicator of cumulative occupational exposure.

To capture patient-reported outcomes (PROs), the SMFDH conducted a survey assessing pain reduction and participant satisfaction to evaluate program feasibility and utility. Anonymous responses were collected and analyzed independently by the SMFDH to avoid linkage or outcome verification bias. The participants received a survey link via text message, with no compensation provided [26]. A total of 148 firefighters responded during 12–18 November 2024, with a response rate of 39.4%. The survey collected demographic information, self-reported numerical rating scale (NRS) pain scores before and after participation, and overall satisfaction (ten items using 0–10 rating scales).

### Statistical analysis

Categorical variables were compared between tenure groups using Pearson’s χ^2^ test. Fisher’s exact test was substituted when any expected cell count was < 5 (e.g., diabetes-medication status). Furthermore, we reviewed the most common diagnosis codes and treatment modalities. To explore differences between groups, we analyzed visit frequency regarding diagnosis categories and medication histories. For survey data, we focused on changes in NRS pain scores (before vs. after intervention) and overall satisfaction (mean ± SD). NRS pain scores were analyzed using two-sided paired t-tests to assess pre-post differences; one-sided tests were avoided despite the reduction trend. All statistical analyses and visualizations were performed in R (version 4.2.0).

## Results

### Baseline characteristics

Over six months, 376 firefighters voluntarily enrolled and received 808 treatment sessions (Table 1). The largest numbers of participants were from Jungbu (n = 117) and Gwangjin (n = 79), where the on-site clinic was also operated at its affiliated firehouses. Firefighters with longer tenure were significantly more likely to be male, older, and hold higher‐ranking or command-related positions than those with shorter tenure. They also showed a distinct duty profile: longer-tenured personnel were more often assigned to command or driving roles, whereas shorter-tenured firefighters were concentrated in frontline EMS and administrative positions.

**Table 1:** Baseline characteristics of study participants (N = 376)

Category	Specific categories	Tenure ≤ 10 yrs (N = 128)	Tenure > 10 yrs (N = 248)	*p*-value	Participants (N = 376)
Gender	Male	103 (80.5%)	223 (89.9%)	0.016*	326 (86.7%)
	Female	25 (19.5%)	25 (10.1%)		50 (13.3%)
Age group	20–29	24 (18.8%)	0 (0%)	< 0.001*	24 (6.4%)
	30–39	88 (68.8%)	24 (9.7%)		112 (29.8%)
	40–49	14 (10.9%)	91 (36.7%)		105 (27.9%)
	≥ 50	2 (1.6%)	133 (53.6%)		135 (35.9%)
Working duty	Command team	6 (4.7%)	29 (11.7%)	< 0.001*	35 (9.3%)
	Fire suppression	32 (25.0%)	60 (24.2%)		92 (24.5%)
	Driving	13 (10.2%)	54 (21.8%)		67 (17.8%)
	Rescue	9 (7.0%)	11 (4.4%)		20 (5.3%)
	Emergency medical services	16 (12.5%)	6 (2.4%)		22 (5.9%)
	Fire investigation	5 (3.9%)	7 (2.8%)		12 (3.2%)
	Administration	47 (36.7%)	81 (32.7%)		128 (34.0%)
Working stations	Dongdaemun	13 (10.2%)	38 (15.3%)	0.159	51 (13.6%)
	Gangdong	16 (12.5%)	47 (19.0%)		63 (16.8%)
	Gwangjin	26 (20.3%)	53 (21.4%)		79 (21.0%)
	Songpa	47 (36.7%)	70 (28.2%)		66 (17.6%)
	Jungbu	26 (20.3%)	40 (16.1%)		117 (31.1%)
Working position	Firefighter	28 (21.9%)	1 (0.4%)	< 0.001*	29 (7.7%)
	Senior firefighter	66 (51.6%)	2 (0.8%)		68 (18.1%)
	Fire sergeant	28 (21.9%)	65 (26.2%)		93 (24.7%)
	Fire lieutenant	1 (0.8%)	110 (44.4%)		111 (29.5%)
	Fire captain	4 (3.1%)	54 (21.8%)		58 (15.4%)
	(Deputy) fire chief	1 (0.8%)	16 (6.5%)		17 (4.5%)
Medications§	Hypertension	2 (1.6%)	39 (15.7%)	< 0.001*	41 (10.9%)
	No hypertension	126 (98.4%)	209 (84.3%)		335 (89.1%)
	Diabetes	1 (0.8%)	10 (4.0%)	0.107	11 (2.9%)
	No diabetes	127 (99.2%)	238 (96.0%)		365 (97.1%)
	Hyperlipidemia	2 (1.6%)	23 (9.3%)	0.009*	25 (6.6%)
	No hyperlipidemia	126 (98.4%)	225 (90.7%)		351 (93.4%)
	Other medications	12 (9.4%)	32 (12.9%)	0.398	44 (11.7%)
	No other medications	116 (90.6%)	216 (87.1%)		332 (88.3%)
	Any medication	14 (10.9%)	84 (33.9%)	< 0.001*	98 (26.1%)
	No medications	114 (89.1%)	164 (66.1%)		278 (73.9%)
History of medical diagnosis §	Cervical herniation	25 (19.5%)	64 (25.8%)	0.201	89 (23.7%)
	No cervical herniation	103 (80.5%)	184 (74.2%)		287 (76.3%)
	Lumbar herniation	67 (52.3%)	126 (50.8%)	0.828	193 (51.3%)
	No lumbar herniation	61 (47.7%)	122 (49.2%)		183 (48.7%)
	Rotator cuff syndrome	4 (3.1%)	13 (5.2%)	0.500	17 (4.5%)
	No rotator cuff syndrome	124 (96.9%)	235 (94.8%)		359 (95.5%)
	Any diagnosis	79 (61.7%)	172 (69.4%)	0.166	251 (66.8%)
	No diagnosis	49 (38.3%)	76 (30.6%)		125 (33.2%)

^*^P < 0.05; Pearson’s χ^2^ test for all categories except Diabetes, for which Fisher’s exact test was used

^§^Participants may have multiple medications and diagnoses, but these diagnosis variables were binary (presence vs. absence)

The use of medication for hypertension and hyperlipidemia was significantly higher in the longer-tenured group. In contrast, the prevalence of common musculoskeletal diagnoses (e.g., cervical or lumbar disc herniation and rotator cuff disorders) did not differ significantly between tenure groups. Cumulative service length was not associated with higher documented MSD rates in this sample. Overall, tenure was primarily associated with demographic, occupational, and medication related differences rather than baseline MSD pathology in this population.

### Service utilization

**Fig. 1:**
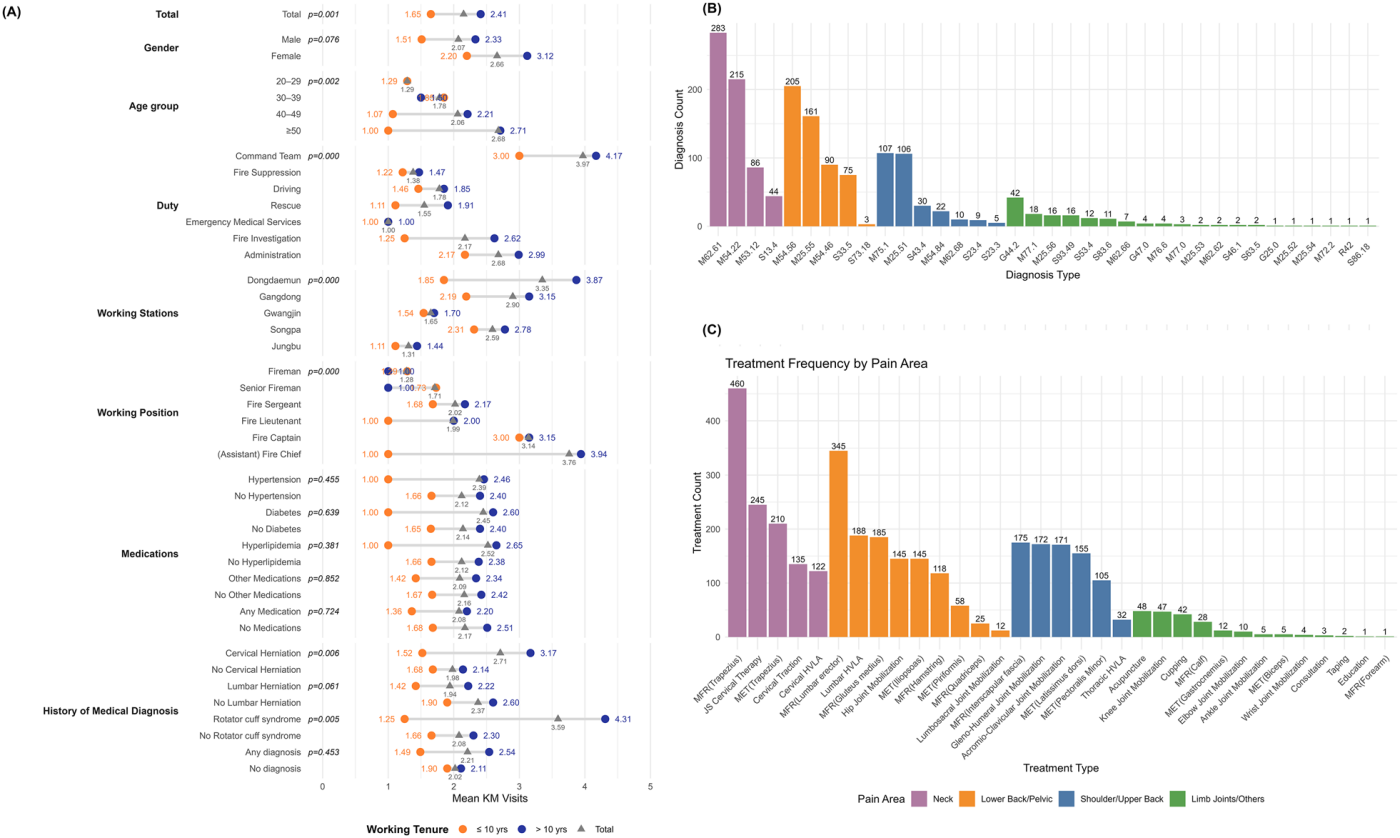
Analysis of clinical records (N = 808; multiple responses allowed). **A** Frequency of ICD-10 diagnoses. **B** Treatment modalities

The average number of visits by category is presented in Fig. 1A (see Supplement 1). As the outcome variable reflects visit counts rather than medical demand, these findings should be interpreted as utilization patterns. Generally, firefighters with more than 10 years of service made more KM visits than their less-tenured peers. By duty assignment, longer-tenured firefighters had more visits than shorter-tenured firefighters across all roles, except in EMS, where all personnel had only one visit. By diagnosis, firefighters with pre-existing cervical disc herniation or rotator cuff disorders also showed significantly higher KM clinic utilization (*p* < 0.05).

Figure 1B presents the distribution of diagnoses and treatment techniques from 808 clinical sessions. Each ICD-10 code is defined in Supplement 2. The most common pain areas were the neck, lower back, shoulder, and limb joints, with corresponding ICD-10 codes primarily falling under the M (musculoskeletal) and S (sprain/strain) categories. Although prior health screenings indicated a history of disc herniation, broader codes such as brachial plexus disorders (M53.12) or sciatica (M54.46) were used to diagnose trunk pain and relevant radiculopathy while avoiding potential diagnostic misclassification. An average of two ICD-10 codes per session were recorded, reflecting combined chief complaints.

In Fig. 1C, treatment patterns closely reflected the distribution of pain areas. Among 808 sessions, the majority (94.8%, n = 766) involved manual therapies, as these were less disruptive during emergency dispatch and required minimal post-treatment downtime. On average, more than four manual techniques were applied per session to address patients’ combined complaints. The remaining 42 sessions consisted of acupuncture, wet cupping, or consultation. In addition to commonly used techniques such as MFR, MET, and joint mobilization, JS cervical therapy—a soft cervical release technique unique to Chuna manual therapy—was applied [27]. Overall, manual therapies predominated due to their logistical convenience within the fire station setting.

### Survey outcomes

Table 2 summarizes the survey respondents’ profiles. The response rate among longer-tenured firefighters was 35.5% (88/248), compared with 46.9% (60/128) for shorter-tenured firefighters. At Gwangjin Station, active promotion yielded the highest station-specific response rate (73.4%; 58/79), whereas Jungbu Station recorded the lowest rate (20.5%). Although Jungbu Station had the largest number of participants (n = 117), most participants attended only once, which corresponded to the lowest survey response rate. By duty type, personnel in EMS (63.6%) and Fire Investigation teams (50%) showed relatively high response rates.

**Table 2:** Baseline characteristics of survey participants (N = 148)

Category	Specific categories	Tenure ≤ 10 yrs (N = 60)	Tenure > 10 yrs (N = 88)	*p*-value	Participants (N = 148)
Gender	Male	43 (71.7%)	75 (85.2%)	0.070	118 (79.7%)
	Female	17 (28.3%)	13 (14.8%)		30 (20.3%)
Age group	20–29	7 (11.7%)	0 (0.0%)	< 0.0001*	7 (4.7%)
	30–39	45 (75.0%)	4 (4.5%)		49 (33.1%)
	40–49	8 (13.3%)	45 (51.1%)		53 (35.8%)
	≥ 50	0 (0.0%)	39 (44.3%)		39 (26.4%)
Working duty	Command team	3 (5.0%)	8 (9.1%)	0.004*	11 (7.4%)
	Fire suppression	18 (30.0%)	43 (48.9%)		61 (41.2%)
	Rescue	2 (3.3%)	4 (4.5%)		6 (4.1%)
	Emergency medical services	12 (20.0%)	2 (2.3%)		14 (9.5%)
	Fire investigation	2 (3.3%)	4 (4.5%)		6 (4.1%)
	Administration	23 (38.3%)	27 (30.7%)		50 (33.8%)
Working stations	Dongdaemun	3 (5.0%)	18 (20.5%)	0.039*	21 (14.2%)
	Gangdong	8 (13.3%)	13 (14.8%)		21 (14.2%)
	Gwangjin	23 (38.3%)	35 (39.8%)		58 (39.2%)
	Songpa	12 (20.0%)	12 (13.6%)		24 (16.2%)
	Jungbu	14 (23.3%)	10 (11.4%)		24 (16.2%)
Working position	Firefighter	7 (11.7%)	0 (0.0%)	< 0.0001*	7 (4.7%)
	Senior firefighter	36 (60.0%)	0 (0.0%)		36 (24.3%)
	Fire sergeant	14 (23.3%)	30 (34.1%)		44 (29.7%)
	Fire lieutenant	1 (1.7%)	35 (39.8%)		36 (24.3%)
	Fire captain	2 (3.3%)	20 (22.7%)		22 (14.9%)
	(Deputy) fire chief	0 (0.0%)	3 (3.4%)		3 (2.0%)
Chief complaints **§** (pain area)	Neck	38 (63.3%)	34 (38.6%)	0.241	72 (48.6%)
	Lower back/Pelvic	29 (48.3%)	46 (52.3%)		75 (50.7%)
	Shoulder/upper back	21 (35.0%)	34 (38.6%)		55 (37.2%)
	Limb joints	12 (20.0%)	17 (19.3%)		29 (19.6%)
	Others	1 (1.7%)	5 (5.7%)		6 (4.1%)
Treatments **§**	Manual treatment (Chuna)	53 (88.3%)	76 (86.4%)	0.080	129 (87.2%)
	Acupuncture	5 (8.3%)	11 (12.5%)		16 (10.8%)
	Cupping	1 (1.7%)	7 (8.0%)		8 (5.4%)
	Consultation/education	9 (15.0%)	6 (6.8%)		15 (10.1%)
	Exercise	7 (11.7%)	17 (19.3%)		24 (16.2%)
	Others	7 (11.7%)	3 (3.4%)		10 (6.8%)

^*^P < 0.05; Pearson’s χ^2^ test was used for gender and working station. For all other categories, Fisher’s exact test was applied

^§^Respondents may have multiple complaints and treatments. The results for these two variables using Fisher’s exact test may be limited in accuracy due to the violation of the independence assumption

**Fig. 2:**
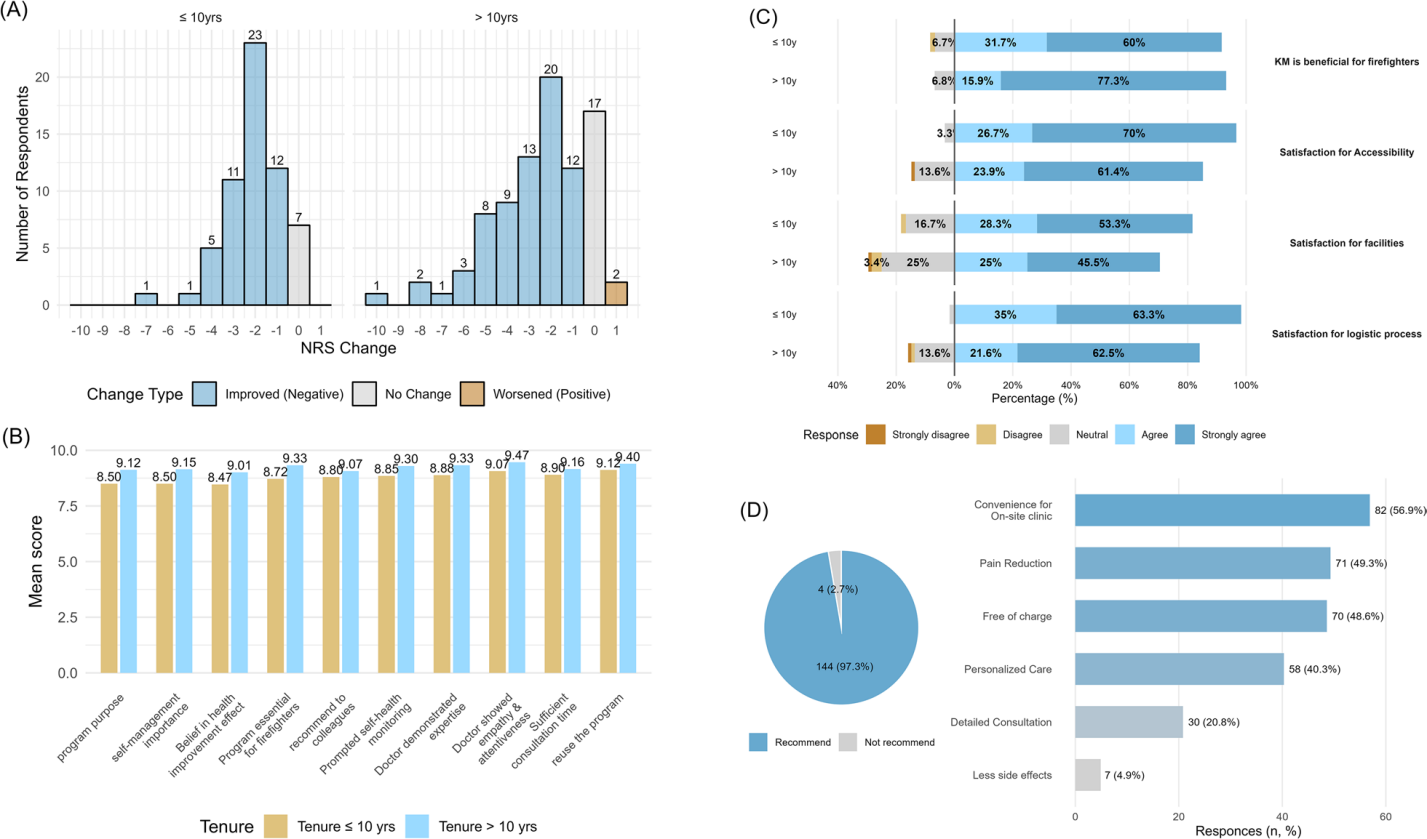
Analysis of survey responses (N = 148). **A** Pre–post comparison of NRS pain scores. **B** Satisfaction ratings (0–10 scale) for clinical service quality. **C** Satisfaction ratings (Likert scale) for administrative processes, accessibility, and application system. **D** Willingness to recommend the service and cited reasons

Figure 2A shows that 82.4% (n = 122) of respondents reported a reduction in pain on the Numerical Rating Scale (NRS). Paired t-tests revealed a mean NRS reduction of 2.45 points (95% CI 1.99–2.91) for longer-tenured firefighters and 2.05 points (95% CI 1.71–2.39) for shorter-tenured firefighters. Among shorter-tenured firefighters, 7 of 60 (11.7%) reported no improvement, compared with 17 of 88 (19.3%) in longer-tenured firefighters. Two longer-tenured respondents reported an increase in pain severity. Across the ten satisfaction and perception items, the overall mean score was 9.05 (SD 1.28). As shown in Fig. 2B, longer-tenured firefighters consistently rated each item higher than shorter-tenured firefighters (9.23 vs. 8.78). Both groups assigned the highest ratings to “intention to reuse the program” and “KM doctor’s attitude and empathy.” On the other hand, “belief in long-term health improvement effect” received the lowest ratings from both groups.

Figure 2C shows similar patterns; longer-tenured firefighters were more likely to report that KM services could contribute to firefighters’ health. Conversely, shorter-tenured firefighters reported higher satisfaction with accessibility and logistical processes, which may reflect greater familiarity with online service applications. Longer-tenured firefighters also expressed lower satisfaction with facilities, which may reflect their broader prior experience with KM services. As shown in Fig. 2D, 144 respondents (97.3%) indicated that they would recommend the service to other firefighters. The most frequently cited reasons included the convenience of the on-site clinic, perceived pain reduction, and the fact that the service is provided free of charge.

To improve the program operation, open-ended responses (n = 50) revealed several suggestions. The most frequent request (n = 11) was to increase the frequency of clinic visits, considering the firefighters’ rotating three-shift schedules. A few respondents also recommended expanding services to additional firehouses and to personnel on external duty. Some comments mentioned equipment upgrades, such as electronic medical record systems and spinal therapy tables. Nevertheless, nearly half of the open responses (n = 24) expressed satisfaction and gratitude, highlighting the program’s convenience, perceived benefits, and the provider’s empathy.

## Discussion

### Summary of findings

Over the course of 808 treatment sessions delivered to 376 firefighters, manual therapy constituted the majority of interventions (94.8%). The utilization patterns varied by tenure and duty type. Firefighters with more than 10 years of service showed higher visits across most subgroups. All EMS personnel attended a single session, whereas repeat visits were observed among firefighters in other duty groups. Participants with prior cervical disc herniation or rotator cuff disorders also demonstrated higher utilization.

In the anonymous survey, 82.4% of respondents reported reduced pain scores and high satisfaction. Differences by tenure were observed, with shorter-tenured firefighters reporting greater reductions but lower satisfaction compared with longer-tenured personnel. These findings should be interpreted cautiously given the retrospective design and absence of a comparison group. Overall acceptability remained high, supporting the feasibility of the program.

### Comparison with previous studies

Previous Korean research on firefighter health has primarily focused on psychological stress and mental health interventions [10]. In contrast, relatively few studies have examined access to musculoskeletal care or physical rehabilitation services. One study using the National Health Insurance Service data highlighted the importance of monitoring MSD risk among firefighters, but focused mainly on descriptive analyses of prevalence and medical expenses rather than intervention or service delivery [28]. International studies have emphasized financial coverage [29] and familiarity with the occupational demands of firefighting [30] as important factors influencing care-seeking. However, most of these studies did not examine access to musculoskeletal care or the implementation of on-site rehabilitation services.

The present program aligns with broader evidence suggesting that in-house clinics facilitate earlier intervention, benefiting employees through improved recovery and employers through sustained productivity [31–33]. For example, prior research on on-site services has shown that such clinics reduce absenteeism, enhance access to care, and contribute to overall organizational efficiency [34, 35]. Unlike programs limited to checkups or referrals, our model directly provided manual therapies to workers identified as high-risk for MSD.

Beyond organizational metrics, more patient-level evidence is needed to determine whether manual therapy benefits high-risk workers. Prior studies have examined the utility of manual therapy among manual workers. For instance, a U.S. observational study reported that early manual therapy reduced rehabilitation time and overall medical costs in occupationally injured workers [36]. Other studies have also evaluated the role of manual therapies as a key modality for pain relief among high-risk workers [37, 38]. Still, there remains limited evidence on the specific techniques [39], cost-effectiveness [40], and clinical outcomes [41] of manual therapy programs in occupational settings. Importantly, these manual therapy studies were largely conducted in the context of off-site service delivery, rather than in on-site workplace settings.

### Strengths and limitations

Delivering on-site manual therapy addresses shift-related access barriers that off-site models leave unresolved. In addition, the study documented the specific manual techniques applied and their associated clinical responses. The practitioner recorded both the manipulative techniques and the patients’ chief complaints. In Korea, firefighters undergo mandatory annual medical examinations, including MRI and CT scans [42]. Thus, their existing diagnoses such as disc herniation or rotator cuff tears could be referenced to support clinical decision-making. Standardized PROs (NRS pain scores, satisfaction) complemented clinical histories to describe program-related outcomes. Primary care settings often lack formal patient evaluation systems, so this approach provides an example of how outcome monitoring can be integrated into routine care.

Nonetheless, important sources of bias remain. First, without a control group, we cannot definitively attribute the observed pain reductions to the intervention. These reductions only represent subjective short-term responses that may reflect regression to the mean or natural recovery rather than a specific treatment effect. Second, reliance on self-reported measures introduces the possibility of recall and selection bias. Third, the dataset reflects only one provider’s clinical decisions, which limits generalizability. As manual therapies are skill-dependent, outcomes may have been influenced by provider proficiency. Fourth, only 24% (376 of 2940) of eligible firefighters participated, leaving the findings vulnerable to selection bias. Because visit frequency varied by duty/tenure, differences in treatment exposure could have influenced outcomes.

### Local context

This program also reflects specific institutional and healthcare contexts in Korea. For example, KM doctors receive formal training during their medical education and regulatory recognition to provide manual therapies such as Chuna manual therapy [21, 22]. Since 2019, Chuna manual therapy has been partially reimbursed by the National Health Insurance Service [43]. This insurance policy contributed to increased utilization of manual therapies by KM doctors nationwide (Supplement 3) [44]. Therefore, this policy context may have facilitated implementation of the present manual therapy program.

In several Korean metropolitan jurisdictions, including Seoul, firefighters are mandatorily enrolled in government-funded group insurance that reimburses most medical expenses [18, 45]. As a result, financial barriers to care are relatively limited. Yet, 24-h duty schedules and the requirement to remain at duty stations often constrain access to off-site services during working hours. In this context, the rationale for providing on-site services was to address time- and location-related barriers to care rather than to reduce direct medical costs. Although this institutional arrangement may reduce generalizability to settings without similar employment-based insurance coverage, it is important for interpreting the implementation logic of the program.

### Implications

The aim of this study was to describe an innovative clinical service delivery model, not to prove the program’s clinical effectiveness. In occupational settings characterized by high physical demand, non-pharmacological on-site interventions may offer a practical option for access to care for MSDs. Without interfering with work duties, such an approach may support symptom management while allowing firefighters to maintain routine operational roles [46]. This pilot model may therefore be particularly relevant in environments where time constraints, workload, or resource limitations reduce opportunities to seek off-site treatment. Still, these observations should be interpreted cautiously given the retrospective design and absence of a comparison group.

Utilization patterns varied by tenure. Firefighters with longer service tended to have more visits, whereas shorter-tenured personnel had fewer opportunities to access the program. These differences may reflect differences in scheduling flexibility, job responsibilities, or access to care rather than differences in treatment response. Shorter-tenured firefighters tended to report lower satisfaction, which may relate to fewer visits and limited exposure to the service. Conversely, longer-tenured firefighters reported smaller reductions in pain, possibly reflecting older age and more chronic conditions.

Duty assignment also appeared to influence participation. All EMS personnel attended only one session, whereas repeat visits occurred among firefighters in other duty groups. This may reflect operational constraints related to dispatch frequency and workload rather than reduced need for care. In 2024, 1521 EMS members in Seoul responded to 557,213 calls and 284,266 emergency transfers [3]. A nationwide doctors’ strike in 2024 may have delayed emergency transfers, which in turn increased the workload of EMS [47]. These structural factors may help explain differential access to on-site services across duty groups.

### Future Research

Based on positive feedback from participating firefighters, the SMFDH continued its partnership with the SKMA to provide ongoing on-site KM clinic services. The Seoul Metropolitan Government subsequently increased financial support and expanded the program to 15 fire stations, staffed by three KM doctors [17]. These operational developments indicate institutional interest in station-based services and may support future studies using more rigorous designs and broader outcome measures. In occupational groups with demanding shift schedules and potential barriers to care, such program expansion may provide a practical context for examining the feasibility of on-site services.

However, the present findings should be interpreted cautiously due to the retrospective observational design and absence of a comparison group. Future research should include objective outcome measures, multi-provider involvement, longer follow-up periods, and appropriate comparison groups. Broader access to diagnostic records would enable more targeted clinical decision-making. Longitudinal follow-up and comparisons between participants and non-participants may help clarify clinical and occupational impacts. Such studies will be essential to assess the feasibility of sustainable integration into occupational health systems.

## Conclusion

This study suggests that a six-month manual therapy program for firefighters was feasible and well accepted. The SMFDH initiated an on-site KM clinic program integrated into daily operations without disrupting work schedules. The program showed high utilization across diverse fire station settings, and differences in perceived pain reduction and satisfaction were observed by tenure, with shorter-tenured firefighters reporting greater pain reduction and longer-tenured firefighters reporting higher satisfaction. These findings suggest that work experience may influence how firefighters engage with and experience on-site care.

Although these findings are preliminary, the retrospective design, reliance on self-reported outcomes, regression to the mean, the local context of this program, and the absence of a control group limit causal inference. Nevertheless, the SMFDH approved a twofold budget increase for the subsequent year [17], indicating institutional support for continued implementation. Future controlled trials incorporating objective functional measures and long-term follow-up are warranted to evaluate effectiveness. Given the high prevalence of musculoskeletal disorders among high-risk workers, on-site manual therapies may represent a feasible component of occupational health care owing to their suitability for diverse work environments.

## Supplementary Information

Additional file1 (DOCX 153 kb)

## Data Availability

The de-identified datasets generated and analyzed during the current study are not publicly available due to personal clinical information. Data may be shared by the corresponding author on reasonable request, pending approval from the institutional review board and compliance with data-protection regulations.

## Abbreviations

ICD: International Classification of Diseases
KM: Traditional Korean Medicine
NHIS: National Health Insurance Services
MSD: Musculoskeletal disorder
EMS: Emergency medical services
SMFDH: Seoul Metropolitan Fire & Disaster Headquarters
SKMA: Seoul Korean Medicine Association
TCIM: Traditional Complementary Integrative Medicine
MFR: Myofascial release
MET: Muscle energy technique
HVLA: High-velocity, low-amplitude
PRO: Patient-reported outcome
NRS: Numerical rating scale
