# Supplementary material for: On-site manual therapy for firefighters in Seoul: six-month utilization and outcome from a retrospective service analysis and web-based survey
Source: Chiropr Man Therap. 2026 Apr 17;34:21. doi: 10.1186/s12998-026-00640-4 (PMC13217992; doi:10.1186/s12998-026-00640-4)
Supplement: Supplementary file 1 — Additional file1 (DOCX 153 kb) [file 12998_2026_640_MOESM1_ESM.docx]

**Supplement 1. Average Visit Counts by Variable (Mean ± Standard Deviation)**

| Category | Specific categories | Tenure ≤10 yrs (N=128) | Tenure >10 yrs (N=248) | p-value | Participants (N=376) |
| --- | --- | --- | --- | --- | --- |
| Total | Toal | 1.65 ± 1.43 | 2.41 ± 2.45 | 0.001* | 2.15 ± 2.19 |
| Gender | Male | 1.51 ± 1.28 | 2.33 ± 2.48 | 0.076 | 2.07 ± 2.20 |
|  | Female | 2.20 ± 1.85 | 3.12 ± 2.11 |  | 2.66 ± 2.02 |
| Age group | 20-29 | 1.29 ± 0.91 | NA | 0.002* | 1.29 ± 0.91 |
|  | 30-39 | 1.85 ± 1.62 | 1.50 ± 1.25 |  | 1.78 ± 1.55 |
|  | 40-49 | 1.07 ± 0.27 | 2.21 ± 1.88 |  | 2.06 ± 1.80 |
|  | ≥50 | 1 ± 0 | 2.71 ± 2.88 |  | 2.68 ± 2.87 |
| Working  Duty | Command Team | 3.00 ± 2.53 | 4.17 ± 3.74 | <0.001* | 3.97 ± 3.57 |
|  | Fire Suppression | 1.22 ± 0.66 | 1.47 ± 1.00 |  | 1.38 ± 0.90 |
|  | Driving | 1.46 ± 0.78 | 1.85 ± 1.50 |  | 1.78 ± 1.39 |
|  | Rescue | 1.11 ± 0.33 | 1.91 ± 1.51 |  | 1.55 ± 1.19 |
|  | Emergency Medical Services | 1 ± 0 | 1 ± 0 |  | 1 ± 0 |
|  | Fire Investigation | 1.25 ± 0.50 | 2.62 ± 2.45 |  | 2.17 ± 2.08 |
|  | Administration | 2.17 ± 1.87 | 2.99 ± 2.85 |  | 2.68 ± 2.55 |
| Working Stations | Dongdaemun | 1.85 ± 1.68 | 3.87 ± 3.81 | <0.001* | 3.35 ± 3.50 |
|  | Gangdong | 2.19 ± 1.72 | 3.15 ± 2.40 |  | 2.90 ± 2.27 |
|  | Gwangjin | 1.54 ± 1.21 | 1.70 ± 1.38 |  | 1.65 ± 1.32 |
|  | Songpa | 2.31 ± 2.07 | 2.78 ± 2.71 |  | 2.59 ±2.47 |
|  | Jungbu | 1.11 ± 0.38 | 1.44 ± 1.16 |  | 1.31 ± 0.94 |
| Working  Position | Fireman | 1.29 ± 1.33 | 1 ± NA | <0.001* | 1.28 ± 1.31 |
|  | Senior Fireman | 1.73 ± 1.38 | 1 ± 0 |  | 1.71 ± 1.36 |
|  | Fire Sergeant | 1.68 ± 1.39 | 2.17 ± 1.88 |  | 2.02 ± 1.76 |
|  | Fire Lieutenant | 1 ± NA | 2.00 ± 1.81 |  | 1.99 ± 1.80 |
|  | Fire Captain | 3.00 ± 2.83 | 3.15 ± 3.25 |  | 3.14 ± 3.20 |
|  | (Assistant) Fire Chief | 1 ± NA | 3.94 ± 4.07 |  | 3.76 ± 4.01 |
| Medications § | Hypertension | 1 ± 0 | 2.46 ± 3.21 | 0.455 | 2.39 ± 3.15 |
|  | No Hypertension | 1.66 ± 1.44 | 2.40 ± 2.29 |  | 2.12 ± 2.04 |
|  | Diabetes | 1 ± NA | 2.60 ± 3.24 | 0.639 | 2.45 ± 3.11 |
|  | No Diabetes | 1.65 ± 1.43 | 2.40 ± 2.42 |  | 2.14 ± 2.16 |
|  | Hyperlipidemia | 1 ± 0 | 2.65 ± 4.00 | 0.381 | 2.52 ± 3.85 |
|  | No Hyperlipidemia | 1.66 ± 1.44 | 2.38 ± 2.25 |  | 2.12 ± 2.02 |
|  | Other Medications | 1.42 ± 1.00 | 2.34 ± 2.80 | 0.852 | 2.09 ± 2.47 |
|  | No Other Medications | 1.67 ± 1.47 | 2.42 ± 2.40 |  | 2.16 ± 2.15 |
|  | Any Medication | 1.36 ± 0.93 | 2.20 ± 2.71 | 0.724 | 2.08 ± 2.55 |
|  | No Medications | 1.68 ± 1.48 | 2.51 ± 2.31 |  | 2.17 ± 2.05 |
| History of Medical Diagnosis § | Cervical Herniation | 1.52 ± 1.26 | 3.17 ± 3.23 | 0.006* | 2.71 ± 2.91 |
|  | No Cervical Herniation | 1.68 ± 1.47 | 2.14 ± 2.06 |  | 1.98 ± 1.88 |
|  | Lumbar Herniation | 1.42 ± 1.02 | 2.22 ± 2.41 | 0.061 | 1.94 ± 2.07 |
|  | No Lumbar Herniation | 1.90 ± 1.75 | 2.60 ± 2.49 |  | 2.37 ± 2.29 |
|  | Rotator cuff syndrome | 1.25 ± 0.5 | 4.31 ± 3.66 | 0.005* | 3.59 ± 3.45 |
|  | No Rotator cuff syndrome | 1.66 ± 1.45 | 2.30 ± 2.33 |  | 2.08 ± 2.09 |
|  | Any diagnosis | 1.49 ± 1.15 | 2.54 ± 2.61 | 0.453 | 2.21 ± 2.31 |
|  | No diagnosis | 1.90 ± 1.77 | 2.11 ± 2.02 |  | 2.02 ± 1.92 |

*** P < 0.05; t‑tests were used for binary variables (total visits, gender, each medication status, each diagnosis status), and one‑way ANOVA was applied to multi‑level categories (age group, working duty, station, position).**

**§ Participants may have multiple medications and diagnoses, but these diagnosis variables were binary (presence vs. absence).**

**Supplement 2. Diagnosis statistics**

| Pain area | rank | Diagnosis  ICD-10 Description | | Count |
| --- | --- | --- | --- | --- |
| Neck | 1 | M62.61 | Muscle strain in the shoulder region | 283 |
|  | 2 | M54.22 | Cervicalgia, cervical region | 216 |
|  | 8 | M53.12 | Cervicobrachial syndrome | 86 |
|  | 10 | S13.4 | Sprain and strain of cervical spine | 44 |
| Lower Back/ Pelvic | 3 | M54.56 | Low back pain, lumbar region | 204 |
|  | 4 | M25.55 | Pain in hip joint | 161 |
|  | 7 | M54.46 | Lumbago with sciatica | 90 |
|  | 9 | S33.5 | Sprain and strain of lumbar spine | 75 |
|  | 25 | S73.18 | Sprain and strain of hip, other parts. | 3 |
| Shoulder/ Upper Back | 5 | M75.1 | Rotator cuff syndrome | 107 |
|  | 6 | M25.51 | Pain in shoulder joint | 106 |
|  | 12 | S43.4 | Sprain and strain of shoulder joint | 30 |
|  | 13 | M54.84 | Other dorsalgia, thoracic region | 22 |
|  | 19 | M62.68 | Muscle strain, other | 10 |
|  | 20 | S23.4 | Sprain and strain of ribs and sternum | 9 |
|  | 22 | S23.3 | Sprain and strain of thoracic spine | 5 |
| Limb Joints/ Others | 11 | G44.2 | Tension-type headache | 42 |
|  | 14 | M77.1 | Lateral epicondylitis | 18 |
|  | 15 | M25.56 | Pain in knee joint | 16 |
|  | 16 | S93.49 | Sprain and strain of ankle | 16 |
|  | 17 | S53.4 | Sprain and strain of elbow | 12 |
|  | 18 | S83.6 | Sprain and strain of other and unspecified parts of knee | 11 |
|  | 21 | M62.66 | Muscle strain in the lower leg | 7 |
|  | 23 | G47.0 | Insomnia, unspecified | 4 |
|  | 23 | M76.6 | Achilles tendinitis | 4 |
|  | 25 | M77.0 | Medial epicondylitis | 3 |
|  | 27 | M25.53 | Pain in wrist joint | 2 |
|  | 27 | M62.62 | Muscle strain in the upper arm | 2 |
|  | 27 | S46.1 | Injury of muscle and tendon of long head of biceps | 2 |
|  | 27 | S63.5 | Sprain and strain of wrist | 2 |
|  | 30 | G25.0 | Tremor, unspecified | 1 |
|  | 30 | M25.52 | Pain in the elbow joint | 1 |
|  | 30 | M25.54 | Pain in the wrist joint | 1 |
|  | 30 | M72.2 | Plantar fascial fibromatosis | 1 |
|  | 30 | R42 | Dizziness and giddiness | 1 |
|  | 30 | S86.18 | Injury of other muscle(s) and tendon(s) of posterior muscle group at lower leg level | 1 |

**Supplement 3. Nationwide Utilization of Chuna Manual Therapy, 2019–2024 (Data Source: Health Insurance Review & Assessment Service) §**

**
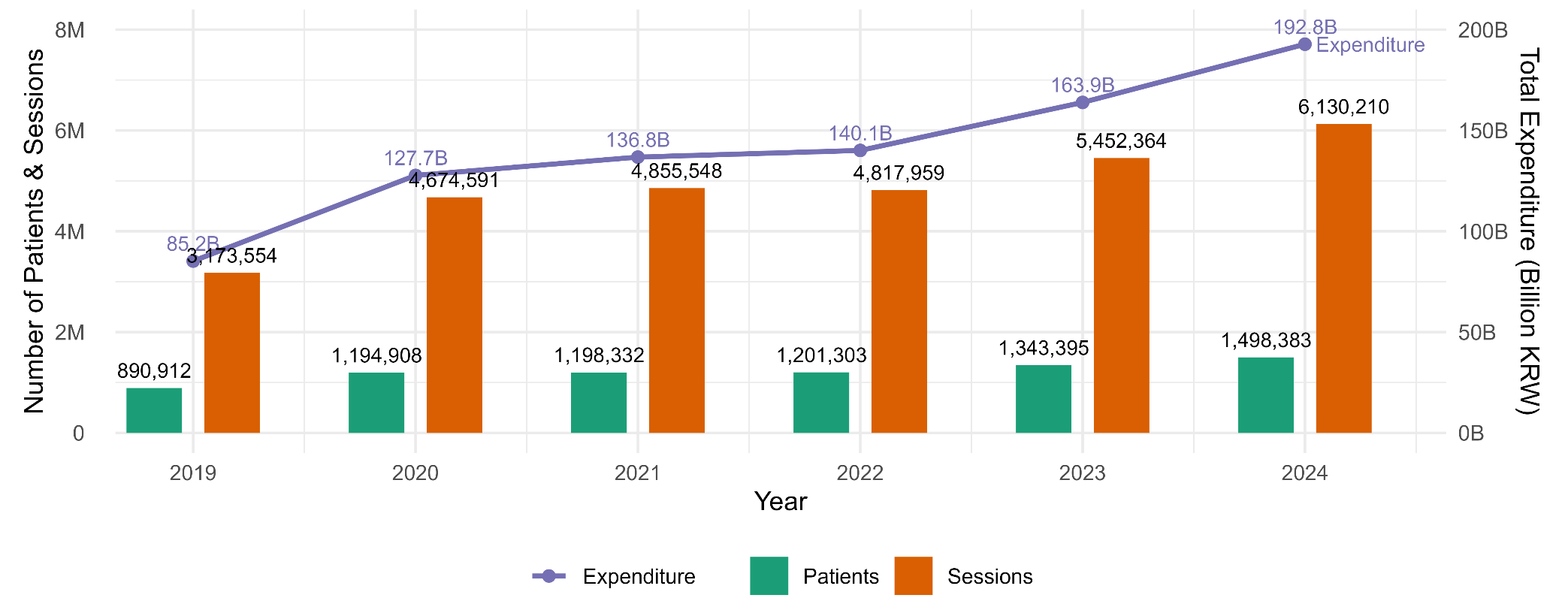
**

**§ Aggregated claims data for procedure codes 4071, 40720, 40721, and 40730**
